# Enhancing the Diagnostic Accuracy of Placental Pathology by Using the Amsterdam Consensus Criteria

**DOI:** 10.7759/cureus.66153

**Published:** 2024-08-05

**Authors:** Murad Alturkustani, Astabraq Alomran, Hind H Al-thomali

**Affiliations:** 1 Pathology, King Abdulaziz University, Jeddah, SAU

**Keywords:** acute chorioamnionitis, chronic villitis, fetal vascular malperfusion, maternal vascular malperfusion, amsterdam criteria, placental pathology

## Abstract

Background and objective

Standardizing placental pathology diagnoses is crucial for improving diagnostic accuracy and clinical communication. The Amsterdam Consensus Criteria were developed to address inconsistencies in diagnosing significant placental pathologies. This study aimed to assess the application and effectiveness of the Amsterdam Consensus Criteria in diagnosing placental pathologies, with a focus on improving the reliability and precision of placental pathology reports.

Methods

A retrospective review of 100 consecutively archived placental pathology samples was performed at a tertiary care hospital. These samples, gathered from January through December 2021, were reassessed according to the Amsterdam criteria. The revised diagnoses were then compared with the original descriptive diagnoses.

Results

Significant changes were noted in all principal diagnoses, including maternal vascular malperfusion (MVM), fetal vascular malperfusion (FVM), chronic villitis of unknown etiology (VUE), and acute chorioamnionitis (ACA). This evaluation led to a recategorization of several cases. Frequently, parenchymal infarcts were reported without adequate information to ascertain their association with MVM. Additionally, there was a noticeable lack of understanding of FVM and VUE among pathologists. ACA was the condition most consistently documented. However, detailed grading and staging were often not included.

Conclusions

Our findings emphasize the need to use standardized diagnostic criteria, such as the Amsterdam criteria, to enhance diagnostic accuracy and facilitate communication between pathologists and clinicians. This will ultimately lead to improved patient care outcomes. It also underlines the necessity of continuous education and calibration for pathologists to mitigate interobserver variability. There is a demand to modify these criteria to ensure universal applicability and relevance in various clinical settings.

## Introduction

The placenta plays a vital role in fetal development and offers insights into both maternal and fetal health through its pathology. However, the assessment of placental pathology faces numerous challenges, including varied diagnoses and interpretations. The critical importance of detailed placental examination cannot be overstated, given its potential to influence neonatal outcomes significantly [1]. Yet, there is a notable decrease in the frequency of pathological examination of placentas [2], particularly in cases with maternal indications. This concern was recently highlighted in a debate where critics questioned the value of placental pathology [3,4], leading the proponents to stress its importance and necessity [5]. This scenario highlights the need for a greater understanding of the causes underlying this trend [6].

The decrease in submission rates and continuous discussion around placental examination relevance can be linked to various factors. These include the complexity of placental pathology reports, as well as the intricate terminology used, which might lead to miscommunication between placental pathologists and clinicians about the reports’ interpretability and clinical relevance [7]. The Amsterdam Consensus Criteria were established as a uniform guideline for identifying critical placental pathologies, including maternal vascular malperfusion (MVM), chronic villitis of unknown etiology (VUE), fetal vascular malperfusion (FVM), and acute chorioamnionitis (ACA). These criteria intend to reconcile clinical and pathological diagnoses by introducing definitive, consistent definitions and sampling strategies for placental lesions [8].

The main goal of this study is to evaluate the application of the Amsterdam Consensus Criteria in diagnosing placental pathologies by comparing the original diagnoses to those made using the Amsterdam criteria. The study seeks to highlight the potential of these criteria to improve the precision and practical usage of placental pathology reports. This, in turn, can foster improved dialogue between pathologists and clinicians, leading to superior patient care. This study’s small sample size is due to it being an initial investigation intended to showcase the impact of using the Amsterdam criteria in a region where many institutions and pathologists continue to depend on descriptive diagnoses.

## Materials and methods

Before undertaking this retrospective study, we obtained ethical approval from the institutional research ethics committee (approval no: 520-23). Given the study’s retrospective design, the need for patient consent was waived. Moreover, there were no direct patient interactions, and strict confidentiality protocols were in place to anonymize patient data. We conducted a retrospective analysis of 100 consecutively archived placental pathology specimens at a tertiary care hospital. Our goal was to curate a sample with minimal bias, representing a wide range of placental pathologies commonly seen in medical practice. 

The inclusion criteria for this study were all placental pathology specimens from singleton pregnancies delivered between January and December 2021, available in the pathology archives. Exclusion criteria included placentas from multiple gestations and placentas that were inadequately preserved. The authors, including a specialist in placental pathology (MA), conducted a reexamination of pathological reports and previously submitted slides to ensure expert analysis and interpretation. This review aimed to identify crucial data such as the gestational age of the fetus at delivery, placental weight, the placental weight reference range based on gestational age, and the umbilical cord coil index.

We contrasted the initial methods of placental examination sampling with the Amsterdam consensus-recommended ones to identify any variances that could affect the accuracy or thoroughness of the original diagnosis. This was essential to shed light on any lapses in the first assessment procedure. The reexamined pathological slides were analyzed using the Amsterdam classification system. This system provides standardized terminology and diagnostic criteria for significant placental pathologies like MVM, FVM, VUE, and ACA. The aim was to harmonize the diagnostic process, thereby facilitating a straightforward comparison between the reevaluated and original diagnoses.

The diagnoses from the reexamination (modified diagnoses) were contrasted with those in the original pathology reports to identify any significant diagnostic differences. The objective was to comprehend the reasoning behind these discrepancies, scrutinizing elements such as sampling errors, interpretative variances, and the original diagnostic criteria’s limitations. This was done to determine the Amsterdam classification system’s influence in enhancing diagnostic accuracy and consistency in placental pathology. This study methodically explored how standardized diagnostic criteria might improve the accuracy and reliability of placental pathology diagnoses, potentially yielding better health outcomes for mothers and infants.

We compared the proportions of cases diagnosed with each placental pathology (MVM, FVM, VUE, ACA, and infarcts) before and after implementing the Amsterdam criteria using chi-square tests and Fisher’s exact tests based on expected contingency table frequencies. Specifically, if all expected cell frequencies were five or above, a chi-square test was applied. The Fisher’s exact test was used where expected frequencies fell below five, ensuring result validity. A p-value <0.05 signified statistical significance. SPSS Statistics (IBM Corp., Armonk, NY) was used for all statistical analyses.

## Results

We rigorously assessed 100 placental samples against the Amsterdam Consensus Criteria for pathological evaluation. With an array of gestational ages and conditions represented, these samples provide a diverse dataset for analysis. These were collected from individuals at various gestational ages, ranging from 17 to 42 weeks, signifying a broad spectrum of pregnancy stages. However, 11 cases lacked recorded gestational ages in the pathology report or electronic system. After excluding these cases, the average gestational age of the placentas was found to be 32.4 weeks. This variability indicates a comprehensive developmental spectrum in the study, from early gestation to full-term pregnancies. The frequent lack of recorded gestational data underscores the need for improved communication between doctors and pathologists, as accurately documenting gestational age is key for matching histological maturation with expected developmental stages and using correct reference ranges for specific gestational ages.

The pathology reports (Table 1) lacked information on the coil index of the umbilical cord, including details on hypo- or hyper-coiling. The description of the umbilical cord is a crucial feature of FVM (8). The weight of the placental disk was not recorded in seven cases, and only one out of 100 reports included the weight’s percentile reference range. This omission points to an area of potential improvement when using standardized reporting systems.

**Table 1:** Data completeness in placental pathology reporting

Data	Provided	Missing/inadequate	Percentage of completeness
Gestational age	89	11	89%
Placental weight	93	7	93%
Coil index provided	0	100	0%
Umbilical cord sampled	99	1	99%
Membranes sampled	99	1	99%
Adequate disk sampling	98	2	98%

An initial review of 100 cases (Table 2) showed that 62 were recorded as having no significant pathologic changes, using terms suggesting either a normal placenta or one without significant pathologic alterations. However, seven cases were marked by increased intervillous fibrin. Using the Amsterdam criteria for reevaluation, 15 cases were reclassified into major diagnostic categories: six were identified as MVM, five as ACA, three as FVM, and one as VUE. Also, two cases were identified with chronic deciduitis, which is not considered a major category. The outcomes of the chi-square test and Fisher’s exact test are presented in Table 3.

**Table 2:** Initial diagnosis and modified diagnosis following the application of the Amsterdam criteria CA: chorioamnionitis; FVM: fetal vascular malperfusion; MVM: maternal vascular malperfusion; NSP: no significant histopathological changes; VUE: villitis of unknown etiology

Initial diagnosis	Frequency	Amsterdam diagnosis	Frequency
NSP	55	MVM	5
		FVM	3
		CA	5
		Chronic deciduitis	2
		VUE	1
		NSP	39
Placenta with increased intervillous fibrin	7	MVM	1
		NSP	6
Decidual vasculopathy	1	MVM	1
Placenta with infarct	17	MVM	8
		Placenta with infarct	9
CA - no staging	18	CA	19
CA - staged	1		
Bivascular umbilical cord	1	Bivascular umbilical cord	1

**Table 3:** Comparison of initial and modified diagnoses using the chi-square test and Fisher’s exact test

Pathology	Initial diagnoses (n)	Modified diagnoses (n)	P-value (chi-square/Fisher’s exact test)
Maternal mascular malperfusion	0	15	<0.001 (Fisher’s exact test)
Fetal vascular malperfusion	0	3	0.025 (Fisher’s exact test)
Acute chorioamnionitis	19	24	0.217 (chi-square test)
Villitis of unknown etiology	0	1	1.000 (Fisher’s exact test)
Placenta with infarct	17	9	0.083 (chi-square test)

The chi-square test revealed no meaningful distinction for ACA (p=0.217) or placenta with infarct (p=0.083). However, Fisher’s exact test demonstrated significant disparities for both MVM (p<0.001) and FVM (p=0.025). There was an insignificant disparity in the diagnosis of VUE (p=1.000).

**Figure 1:**
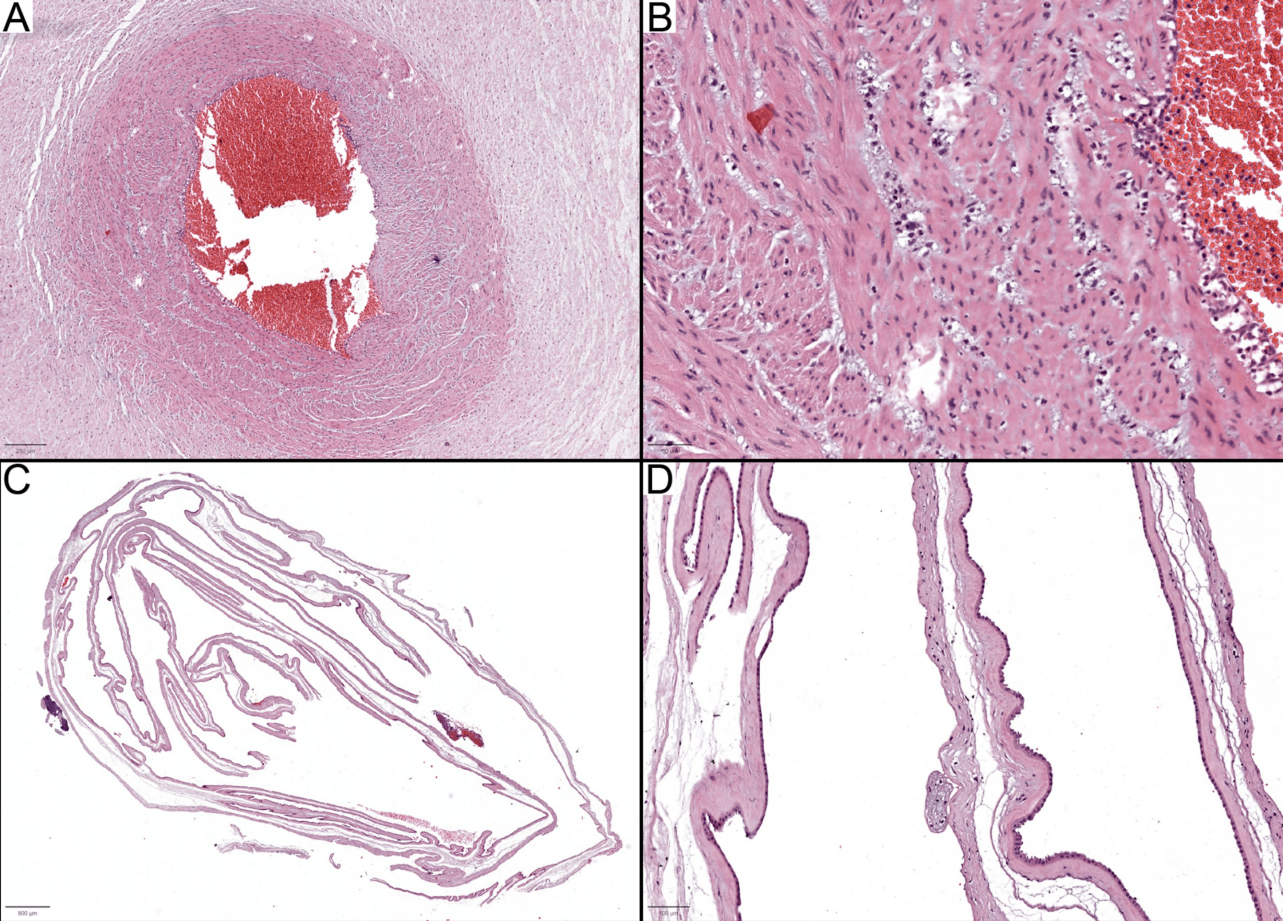
Case 9 with chorioamnionitis and inadequate membrane sampling (A) The umbilical cord reveals neutrophilic infiltration within the vascular walls, a detail potentially overlooked at low magnification. (B) At higher magnification, the presence of neutrophils within the vascular walls is clearly visible. (C, D) Images of the membrane sample, which includes only amnion without chorion, hinder a thorough examination of maternal inflammatory response. The membrane sample is stained with hematoxylin and eosin. Scale bars are as follows: A: 250 μm; B: 50 μm; C: 800 μm; and D: 100 μm

Two cases from the first group with no significant pathological changes are discussed in detail here. Case 9 involved a 33-week gestational placenta, described as mature with a trivascular cord. A detailed examination of the umbilical cord revealed acute inflammation in the vascular walls of both the veins and arteries. This inflammation, which can be easily overlooked on a low-power examination (Figures 1A, 1B), was indicative of funisitis stage 2, grade 1. The membrane roll sample showed the presence of amnion without a chorion (Figures 1C, 1D), hindering the evaluation of the maternal inflammatory response to chorioamnionitis. This case underscores the risk of misdiagnosis and the impact of insufficient membrane sampling on a comprehensive condition assessment.

**Figure 2:**
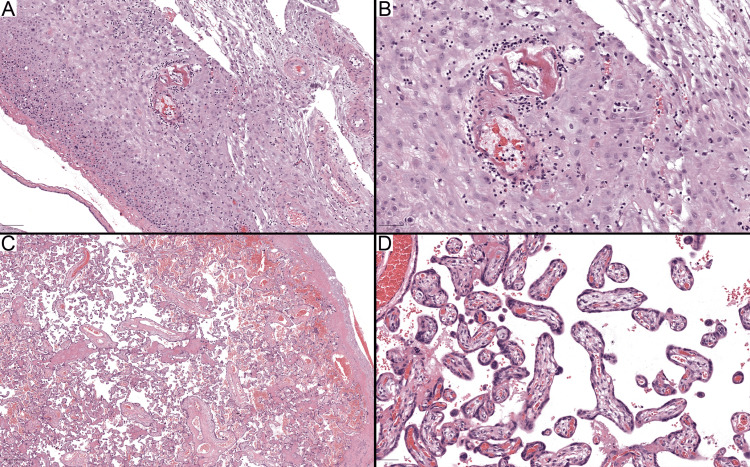
Case 23 with maternal vascular malperfusion (A, B) Decidual vasculopathy is characterized by fibrinoid necrosis and lymphocytic perivascular infiltration around small arterioles in the decidual membrane. (C, D) The depicted chorionic villi are elongated with peripheral capillaries and syncytial knots, indicative of villi maturity typical of term placenta. Stained with hematoxylin and eosin. Scale bars: A and C: 100 μm; B and D: 50 μm

Case 23 involved a 26-week gestation placenta that was originally reported as a mature third-trimester placenta. However, upon reevaluation, the placenta displayed focal decidual vasculopathy, characterized by the fibrinoid necrosis of decidual arterioles surrounded by a lymphocytic infiltrate (Figures 2A, 2B). The morphology of the chorionic villi suggested a mature term placenta (Figure 2C, 2D). Considering the gestational age of 26 weeks, these results pointed towards accelerated villous maturation (AVM), leading to an updated diagnosis of MVM. This case emphasizes the importance of a thorough placental examination and correlating histopathological findings with gestational age for an accurate diagnosis.

In the initial account, 17 samples were vaguely described as having infarcts without a specific diagnosis. Upon further review, eight samples exhibited additional traits consistent with MVM criteria. The remaining nine samples did not show adequate evidence in their sections to conclusively diagnose MVM. This suggests potential sampling inadequacies or the true absence of MVM-specific properties. The Amsterdam Consensus Criteria did not offer further guidance for optimum diagnosis in these cases. Accordingly, we decided to keep the original diagnosis for these samples. However, recent studies have noted that the mere presence of an infarct could suggest MVM - with a 52% probability correlating to adverse maternal obstetrical outcomes [9].

**Figure 3:**
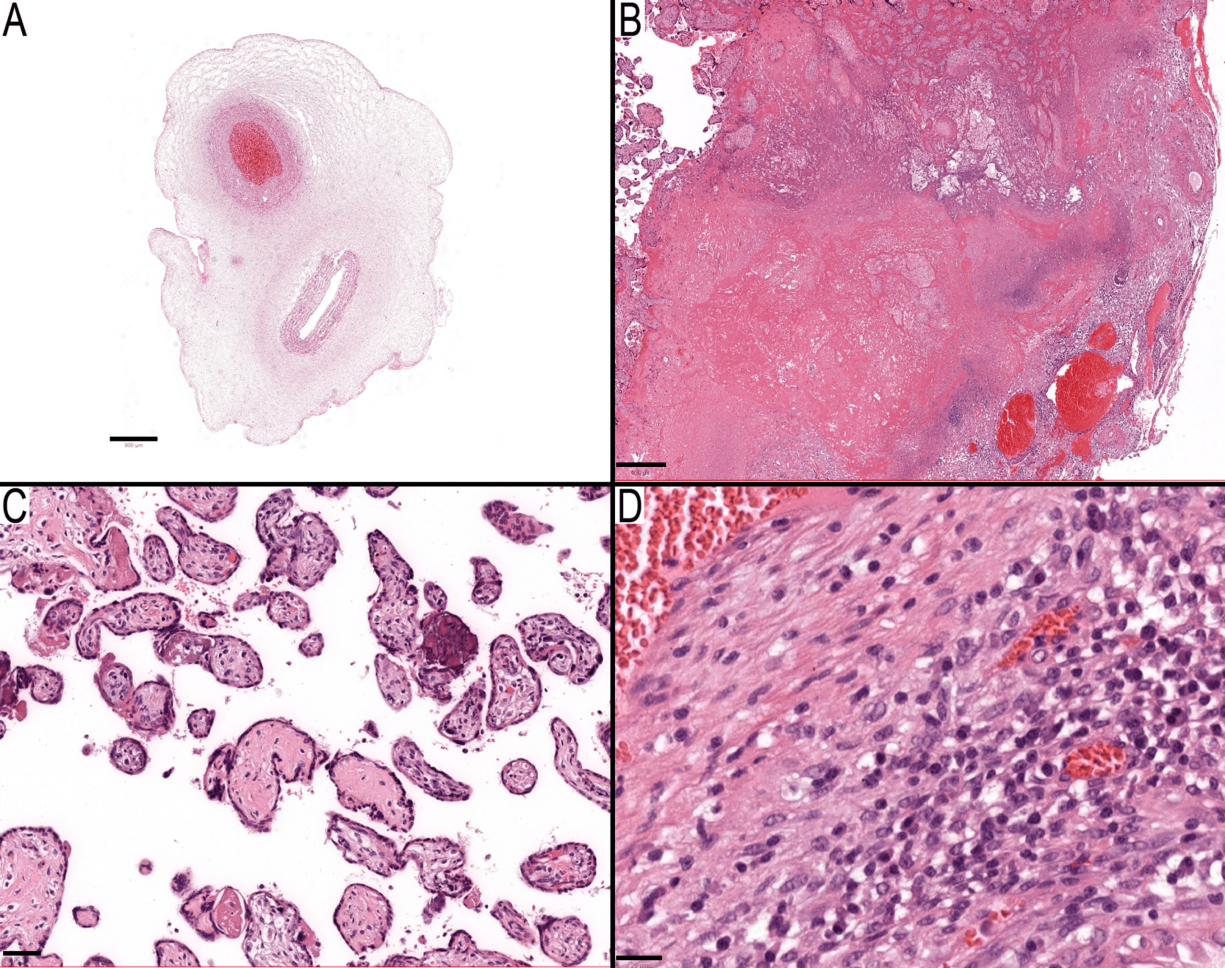
Case involving a bivascular umbilical cord and a case diagnosed as placenta with an infarct (A) Umbilical cord featuring two vessels (artery and vein). (B-D) A case exhibiting maternal vascular malperfusion along with additional pathological features. (B) Infarct hematoma is characterized by a round hemorrhagic focus surrounded by infarcted villi. (C) Scattered avascular villi with no other diagnostic features of fetal vascular malperfusion present. (D) Decidua containing plasma cells, indicative of chronic deciduous. All sections are stained with hematoxylin and eosin. Scale barsL A: 800 μm; B: 400 μm; C: 50 μm; and D: 20 μm

Case 78 features a placenta with a bivascular umbilical cord that did not exhibit any other significant pathology (Figure 3A). Case 83 was characterized by a placenta in which the gestational age was not specified. The final diagnosis was an unqualified placenta with an infarct, but the infarct's location was not described. Pathological reassessment of the provided slide highlighted features of MVM, including an infarct hematoma (Figure 3B), alongside scattered avascular villi in the placenta. However, the density of these villi was insufficient for a diagnosis of FVM (Figure 3C). Furthermore, the presence of plasma cells in the decidua, indicative of chronic deciduitis, was another significant finding (Figure 3D).

The results of this study highlight the importance of exhaustive and meticulous pathological reporting and the usefulness of the Amsterdam Consensus Criteria in improving diagnostic precision for placental pathologies. Our findings indicate that adhering to standard criteria greatly enhances the detection and comprehension of placental diseases, potentially resulting in improved clinical outcomes.

## Discussion

The study highlights a wider issue within placental pathology practice at a tertiary hospital, where differences in examination protocols and diagnostic criteria may obstruct standardization. Despite the existence of consensus criteria for placental pathology, many pathologists still use traditional descriptive diagnoses. Our findings stress the value of standardized criteria in placental pathology assessments over traditional descriptive diagnosis. Using the Amsterdam criteria in such situations promotes a more accurate diagnosis and points out potential underdiagnosis or misclassification in traditional reporting. The need for thorough examination and documentation, as the Amsterdam criteria recommend, is highlighted by the reclassification of cases initially thought to have no significant findings into specific pathological categories. This method could significantly improve the diagnostic usefulness of placental pathology, providing a clearer understanding of maternal and fetal health implications.

In our study, we juxtaposed the traditional descriptive diagnosis with the criteria of the Amsterdam consensus. We frequently observed mentions of parenchymal infarcts in pathology reports in our findings. However, these reports often do not provide sufficient detail to ascertain a definitive diagnosis. Acknowledging a placental infarct is key to diagnosing MVM. Yet, our review found that these reports rarely clarify whether the mentioned parenchymal infarcts aid in an MVM diagnosis, leaving their clinical relevance doubtful. The understanding of these infarcts heavily relies on gestational age; minor peripheral infarcts in a term placenta may be considered normal, unlike those in preterm pregnancies or centrally located infarcts, which bear more weight [8]. The correlation of MVM with adverse results, including fetal growth restriction (FGR), placental abruption [10], intrauterine fetal demise (IUFD) [11], preterm birth (PTB), and a higher risk of cardiovascular diseases for both mother and child, emphasizes the clinical significance of the diagnosis [12].

The existing consensus document discusses a range of pathologies associated with MVM, but further details about necessary diagnostic features are required. Recent research has built on this, incorporating findings such as AVM and distal villous hypoplasia (DVH) as diagnostic indicators and considering villous infarct as a secondary discovery [13]. Our review failed to find any diagnoses of FVM or VUE in the examined cases, highlighting a potential lack of awareness or difficulty among pathologists in identifying these conditions using traditional descriptive diagnoses. Detection of FVM features can be improved with the use of a CD34 immunostain [14]. The Amsterdam consensus outlines the histological features needed for diagnosing FVM and VUE, underscoring the need to understand the reach of these diseases. Clinically, FVM has been associated with neonatal neurological complications such as encephalopathy [15], cerebral palsy [16], stroke [17], and IUFD [18]. While a recent review challenged the link between FVM and encephalopathy, further research is needed to elucidate these associations [19].

Identifying a case of VUE requires a careful review of slides to detect lymphocytic infiltrates in the chorionic villi. VUE is considered a maternal inflammatory immune response to fetal villi, and it has a notable recurrence rate in future pregnancies [20]. It is essential to diagnose VUE as it is associated with FGR [21], cerebral palsy, and IUFD [13]. Thus, its identification is critical. A separate retrospective study on 127 placentas found no significant correlation between the specific characteristics of VUE (such as severity, distribution, or location) and FGR [22]. However, the location of VUE might be tied to worse neonatal outcomes. It implies that any type of villitis could disrupt placental function and impact fetal growth [22].

ACA was the most frequently recorded pathology in our cohort, with diagnosis rates remaining steady (p=0.217), which reflects an increasing awareness among pathologists. However, the reports often did not include detailed grading and staging of both maternal and fetal inflammatory responses. Even though the Amsterdam criteria prioritize identifying ACA, they also provide grading criteria. The clinical diagnosis of ACA is not the same as a pathological diagnosis, and low-stage ACA may not be strongly linked to an infectious cause [23]. Also, a high-stage maternal inflammatory response correlates with bacterial load [24], and the fetal inflammatory response is linked to unfavorable fetal outcomes [25,26]. In particular, high-stage (stage 3) maternal and stage 2 fetal inflammatory responses were associated with germinal matrix hemorrhage-intraventricular hemorrhage [27].

The Amsterdam Consensus Criteria serve as an important reference for placental examination. However, it is pivotal to look at other existing guidelines as well. The College of American Pathologists (CAP) provides comprehensive placental examination guidelines covering maternal, fetal, and placental factors that necessitate a thorough histopathologic evaluation. The CAP guidelines aim to select placentas for examination based on validated indications, offering insights into immediate and future outcomes for the mother and child, as well as identifying risks for prospective pregnancies [28]. Furthermore, using a modified Delphi method, 16 placental pathologists have created specific criteria for placental triage. This stresses the need to examine placentas in circumstances like pregnancy loss, suspected maternal infection, presumed abruption, FGR, and other urgent clinical scenarios [29]. Such evaluations enhance our comprehension of pathophysiology, support practice evaluation, and contribute to the advancement of obstetric and neonatal sciences, ensuring specialized and informed clinical care [29].

It is crucial to understand that many pathological terms and their perceived importance may shift over time. Hence, original disease descriptions might still be accurate. The Amsterdam criteria are particularly effective as a specific diagnostic blueprint. They tie specific diagnoses to certain risk factors and corresponding outcomes of significant clinical importance. Moreover, They can serve as a thorough checklist, encouraging pathologists to explore additional pathological findings [8]. However, keeping abreast of recent research is crucial to maintain the relevance of these diagnoses. For example, a prime feature of FVM, vascular ectasia, is less significant in formalin-fixed placentas, likely due to it being an artifact [30]. Another study proposes making multinucleate giant cells of implantation-type trophoblasts a primary criterion for MVM [31]. Another limitation emerges due to the lack of certain important details from the referring physicians, particularly the omitted gestational age. This data is key for accurately interpreting placental weight and different pathological features. The required information that should be included comprises previous pregnancy outcomes, the reason for referring the placenta to pathology, and any notable maternal or fetal medical history.

This study was limited by inadequate documentation of severe acute respiratory syndrome coronavirus 2 (SARS-CoV-2) infections, with only one case in the cohort explicitly reporting a positive result. The status of the other 99 cases remained unclear, although the placentas were collected during a period of widespread infection. The impact of this infection on placental pathology is still uncertain since various major diagnostic categories were associated with the infection. A previous study analyzed 20 placentas from mothers with the novel coronavirus and identified considerable connections in 10 cases, associating coronavirus disease 2019 (COVID-19) with conditions like FVM or thrombosis [32]. Another study of five placentas from SARS-CoV-2-positive women revealed morphological changes and detected viral RNA in the placenta and umbilical cord tissues. All cases showed MVM and some FVM, with no infants being infected [33].

Other research found features of MVM, including increased syncytial knots in all eight cases studied and increased focal perivillous fibrin deposits in seven cases [34]. A comprehensive review emphasized the ongoing challenges of assessing the impact of maternal SARS-CoV-2 infection on placental pathology, such as the lack of control groups and a focus on third-trimester infections. It recommended more collaboration and larger sample sizes for more reliable findings [35]. However, these issues should not affect the present study, which centers on using the Amsterdam criteria and comparing it with the descriptive diagnosis.

Despite the widespread use and acceptance of the Amsterdam Consensus Criteria in placental pathology, there are still ongoing challenges. One significant issue is the inconsistency among observers [36], emphasizing the need for ongoing education and calibration among pathologists to ensure the uniform application of the criteria. Additionally, while the criteria effectively cover common pathologies, they may still need to expand to include less common but important pathologies fully. This underscores the need for the criteria to continuously evolve, ensuring they stay current and inclusive with the latest pathological research developments.

Another difficulty, highlighted in a recent study of a cohort of 19,027 placentas, involved the significance of overlapping placental pathology patterns and their link to adverse birth outcomes [37]. Notably, findings showed that conditions like MVM, alone or in combination with others like VUE and FVM, considerably increased the likelihood of small for gestational age (SGA) infants and PTB. The researchers then devised a placental phenotypic classification that takes the severity and multiplicity of lesions into account, aiding in clinical assessments and increasing the usefulness of placental pathology findings in research [37].

The study has several limitations, primarily its small sample size of 100 placental specimens, which may have failed to capture the full array of placental pathologies found in wider clinical practice. Also, the study was retrospective in design, depending on archived specimens, and thus may be subjected to biases inherent in sample selection and preservation. Despite these limitations, the study underscores the inconsistency in diagnostic practices among pathologists, underlining the necessity for continued education and standardization to minimize interobserver variability.

## Conclusions

To successfully apply these criteria and report based on them, clinicians should provide essential information and actively liaise with pathologists for clarifications or specific inquiries. This crucial collaboration enhances placental pathology practices, steering them toward maximizing clinical utility and improving patient care outcomes. Our study highlights the potential of the Amsterdam criteria to transform the assessment of placental pathology. We endorse its broad application to unify reporting practices, boost diagnostic precision, and improve clinical results.

Addressing the limitations of these criteria, fostering global collaboration, and ensuring that placental examination practices are guided by the most recent scientific evidence and consensus guidelines represent key steps forward. A collective approach, emphasizing the provision of essential clinical information by referring clinicians and open dialogue with pathologists, will direct placental pathology practice toward considerable advancements.
